# Retrospective Analysis of Cancer Care Performance and Survival Outcome for Nasopharyngeal Carcinoma at a leading Cancer Treatment Centre in Malaysia 2008-2012

**DOI:** 10.31557/APJCP.2019.20.6.1701

**Published:** 2019

**Authors:** Matin Mellor Abdullah, Yoke Ching Foo, Beng Khiong Yap, Catherine May Ling Lee, L P Hoo, Teck Onn Lim

**Affiliations:** 1 *Cancer and Radiosurgery Centre, Subang Jaya Medical Centre, *; 2 *Clin Research Sdn Bhd, Kuala Lumpur, Malaysia. *

**Keywords:** Nasopharyngeal carcinoma, Survival, Outcome, Performance, Measures

## Abstract

**Objective:**

This report focuses on a private medical centre cancer care performance as measured by patient survival outcome for up to 5 years.

**Methods:**

All patients with nasopharyngeal cancer treated at SJMC between 2008 and 2012 were enrolled for this observational cohort study. Mortality outcome was ascertained through record linkage with national death register, linkage with hospital registration system and finally through direct contact by phone.

**Result:**

266 patients treated between 2008 and 2012 were included for survival analysis. 31% of patients were diagnosed with Early NPC Cancer (Stage I or II), another 44% with Locally Advanced Cancer (Stage III) and 25% with late stage IV metastatic cancer. 2%, 27% and 67% had WHO Class I, II and III NPC respectively. The overall survival at 5 years was 100% for patients with Stage I disease, 91% for Stage II disease, 72% for Stage III disease, and decreasing to 44% for Stage IV disease. Overall survival at 5 years for all stages was 73%.

**Conclusion:**

SJMC is among the first hospitals in Malaysia to embark on routine measurement of the performance of its cancer care services and its results are comparable to any leading centers in developed countries.

## Introduction

Nasopharyngeal carcinoma (NPC) arises in the nasopharnyx, a cuboidal chamber behind the nasal cavity. Although it is rare in most parts of the world, it exhibits a distinct geographical and racial distribution that is common in South China and South Eastern Asia (Petersson, 2015). Currently WHO classifies nasopharyngeal carcinoma (NPC) into 2 separate categories: Type 1 keratinising (K) carcinoma (squamous cell carcinoma; SCC) and Type 2 non-keratinising (NK) carcinoma which can be subdivided into including differentiated and undifferentiated subtypes.

NPC has a high incidence in South East Asia especially among the Chinese, of South China origin and is mainly attributed to the non-keratinising subtype (Petersson, 2015). An association with Epstein Barr virus (EBV) is also well established in these populations, where there is also an increased tendency to metastasize to regional lymph nodes and distant sites (Colaco et al., 2012).

In Malaysia, NPC is the fifth most common cancer in the Malaysian population and third most common in males. The lifetime risk for males was 1 in 143 and for females was 1 in 417. The incidence in males increased at the age of 25 years old and peaked at the age of 65 years old (Tamin et al., 2011).

NPC is predominant among Chinese (49%), followed by the natives of Sabah and Sarawak (28%) and Malay (22%). In Sarawak, high incidence of NPC is reported among Bidayuh (48.4%) (Tiong and Selva, 2005). 

Various studies have shown an encouraging trend, with steady improvement in treatment outcomes of NPC (Huang et al., 2015). Two recent advances in NPC treatment, intensity-modulated radiation therapy (IMRT) and concurrent use of chemotherapy may have contributed to this improvement. However, data regarding NPC survival and associated prognostic factors from numerous participants in a nationwide database are scarce.

Subang Jaya Medical Centre’s (SJMC) cancer care services, and specifically nasopharyngeal cancer care, is the focus of this report. We have previously reported on SJMC’s breast cancer care performance for survival outcome and process measures (Abdullah et al., 2014). The present report focuses on SJMC’s care performance for NPC as measured by patient survival outcome for up to 5 years. Cancer survival is a key index of the overall effectiveness of health services in the management of patients with cancer. We present our single institution data (SJMC) on patients diagnosed with NPC and treated over 5 years from 2008-2012 as well as our experience with regards to NPC Cancer Care performance measures.

## Materials and methods

This was a retrospective study based on medical records review. The protocol was approved by Ministry of Health Medical Research and Ethics Committee (Approval number P11-550).

*Patients*

In this retrospective study, the patients consists of Malaysians with pathologically confirmed Nasopharyngeal Carcinoma diagnosed between 2008 and 2012, and treated with at least one treatment modality at SJMC. A total of 278 cases were identified through the hospital register as well as chemotherapy and radiotherapy records. Case ascertainment was independently verified to be complete (100%). The 2008-2012 period was selected in order to best balance an appropriate indication of prognosis with the need to identify benefit from contemporary treatment regimens

*Data collection and definitions*

Data were extracted from patients’ medical and histopathology (HPE) reports by trained data collectors. Demographic data extracted included age, sex, race and nationality; tumour characteristics included histologic type, grade, location, extent, and size; lymph node and distant organ metastases. Staging of disease was carried out based on TNM staging. TNM stage I or II disease were considered early cancer, stage III locally advanced cancer and stage IV metastatic cancer. Based on the HPE report, tumour histology was categorised as keratinising or non-keratinising according to WHO classification (2005). 

There are no published healthcare performance measures in routine use for NPC care. We therefore adopted and modified the performance measures used for other solid cancers that were developed by Quality Oncology Practice Initiative (QOPI) (Campion et al., 2011) and American Society of Clinical Oncology/ National Comprehensive Cancer Network (ASCO-NCCN) (Desch et al., 2008), while taking into account clinical practice guidelines (CPG) for NPC (Ministry of Health, 2016).

*Treatment*

The most common radiotherapy fractionation used during the course of Tomotherapy was 70Gy in 35 fractions once daily with an overall treatment time (OTT) of 7 weeks. Average IMRT dose used was 62.9Gy in 34 fractions once daily with an overall treatment time (OTT) of 7 weeks. The radiotherapy dose as well as decision to treat with neoadjuvant and/or concurrent chemo-radiotherapy was at the discretion of the treating clinical oncologist. All patients included in this study underwent 3D CT simulation and had at least 3D Conformal Radiotherapy. In the event, 16 patients (6%) had treatment with 3D Conformal Radiotherapy, 93 patients (36%) had treatment with LINAC Intensity Modulated Radiation Therapy and 151 patients (58%) had treatment using Tomotherapy (Helical Intensity Modulated Radiation Therapy). Where neo-adjuvant chemotherapy was given, Gemcitabine + Platinum or Paclitaxel + Platinum based chemotherapy agent combinations were given 3 weeks apart. Patients receiving concurrent chemo-radiotherapy received weekly Cisplatin (40mg/m2) during radiotherapy treatment.

*Mortality ascertainment and imputation*

Complete and accurate ascertainment of mortality outcome among study patients is necessary to minimize bias in estimating cancer survival outcome. We followed a rigorous procedure described below to ensure this.

1. Case ascertainment was initially independently verified to be complete (100%). This was to avoid exclusion of deceased patients especially those who die soon after diagnosis. 

2. Mortality outcomes were noted during data extraction for the study (19 deaths identified). 

3. All cases enrolled were matched in 2014, based on their names and national identity card number against the national mortality database maintained by the National Registration Department to ascertain their mortality outcome (total 54 deaths identified).

4. Remaining cases were matched based on their names and hospital number against the hospital register (which records all visits to the hospital). Patients who had a visit after the end of the study period (31 Dec 2014) were considered alive (127 ascertained alive). 

5. A sample of the remaining cases -24 patients (69%) with Stage I or II and 100% (49 patients) of cases with Stage III or IV were contacted by phone or home visit to ascertain mortality outcomes. Two (10%) patients out of 19 with Stage I or Stage II had died, likewise for one (3%) out of 28 Stage III and two (23%) out of eleven Stage IV patients. 

6. For the purpose of survival analysis, we therefore assumed all patients with Stage I or Stage II who were uncontactable (6 cases) to have a death risk of 10%. We further assumed all remaining uncontactable patients with Stage III (3 cases) or IV (2 cases) to be dead. Thus, any bias in the survival estimates arising from missing information on mortality outcome is conservative (that is, the survival estimates can only be worse than they actually are).

*Independent data audit*

HPE reports were retrieved for all patients enrolled to verify tumor diagnosis and characteristics. In addition, patients’ demographic and treatment data were also subjected to independent data verification against source documents on site. The accuracy of the collected data with respect to demographics, radiotherapy and chemotherapy were all > 95%.

*Statistical methods*

Continuous variables are described by summary statistics such as mean, median, and standard deviation and categorical (nominal/ordinal) variables, by the frequencies of each category.

For cancer survival outcome performance, results are expressed as overall survival and relative survival. Relative survival is the ratio of the survival observed in the study patients and the survival that would be expected if they had experienced only the background mortality (all-cause death rates) of the general population of the same age, sex and ethnicity. It shows the extent to which cancer shortens life compares to the general population.

Age standardised five-year relative survival is used for comparison of survival outcome between this study population and other centres’ or registry populations. Age standardised rate refers to the rate that would be observed if the patient populations compared had the same age structure as an external standard population, in this case, the International Cancer Survival Standard (Corazziari et al., 2004). Age standardisation allows comparison of results between jurisdictions or countries. Multivariable Cox regression is used to estimate the effects of covariates on survival outcome. The level of significance was set at 0.05. This analysis included only patients with non-keratinizing NPC (7 with keratinizing or other NPC excluded) who have received Radiotherapy at SJMC (3 who received palliative chemotherapy only excluded).

## Results

A total of 278 patients who were potentially eligible for inclusion in this study were identified to have NPC through the hospital register as well as chemotherapy and radiotherapy records. A total of 12 patients were excluded from analysis; three because they have recurrent cancers, 1 for pre-cancer, 5 were Medical tourists (foreigners), and 3 were patients from another centre whose radiotherapy was outsourced to SJMC. The final analysis set thus has 266 subjects.

Thus the final sample size was 266 subjects. These 266 subjects have data on at least one treatment episode. A treatment episode refers to the specific treatment modality (radiotherapy, chemotherapy etc) provided by a single centre for an individual patient. Minimal data required to define a treatment episode is Centre name, Patient name, date diagnosis, date start treatment, basic details to describe the treatment

*Demographics*

Table 1 shows the patients’ demographic and tumour characteristics. The mean and median age was only 51 years (27 years to 86 years); 46% was aged <50 years, 93% was Chinese and 56% resided in Klang Valley or Selangor. 31% were women and 69% were men. 45% paid for their care out-of-pocket (OOP) and 51% had their care financed by insurance or their employer. 

*Stage at Presentation & Histological Subtype*

31% of patients (82 patients) were diagnosed with Early NPC Cancer (Stage I or II), another 44% (118 patients) with Locally Advanced Cancer (Stage III) and 25% (66 patients) with stage IV metastatic cancer. Five patients (2%) were found to have keratinizing SCC (WHO type I), 74 patients (28%) non-keratinizing differentiated (WHO type II) and 185 patients (70%) non-keratinizing undifferentiated (WHO type III). Data was not available for 2 patients in the absence of HPE reports. For patients first presenting at SJMC, it took a median of 7 days to arrive at a diagnosis of NPC.

*Treatment*

Of the 266 patients who completed treatment with curative intent, 263 (99%) received radiotherapy and 234 (88%) received radiotherapy with chemotherapy. 33 (12%) patients received radiotherapy alone, 174 (65%) received concurrent chemo-radiotherapy while the remaining 18 (7%) patients had neo-adjuvant chemotherapy followed by concurrent chemo-radiotherapy 99% of patients have had Radiotherapy, with increasing trend towards utilization of IMRT and Tomotherapy in more recent years (91% used IMRT-Tomotherapy in 2012, while 88% had IMRT-Linac in 2008). 81% of patients have had Cisplatin chemotherapy, and 18% had Gemcitabine. 

Among the patients who received chemotherapy treatment, 75% of the patients had concurrent radiotherapy with chemotherapy, 17% of the patients had neo-adjuvant chemotherapy with radiotherapy and 8% of the patients had neo-adjuvant chemotherapy follow by concurrent radiotherapy with chemotherapy.

Duration from diagnosis to first treatment was performed in median of 21 days. Median duration to initiate any treatment for Stage III/IV was only 21 days while for Stage I/II was 23 days. Median Duration from diagnosis to starting Radiotherapy for advanced disease was 17 days with a mean of 24 days. 

The planned overall treatment time (OTT) was 7 weeks and 69% were able to complete their Radiotherapy within this time period. Median OTT was 48 days while mean OTT was 47 days. Interruptions in RT causing a prolonged treatment time have been reported to be detrimental for local control and survival in NPC. In our centre, the interruptions were mostly attributed to public holidays.

*NPC Care Process performance*

Table 2 summarizes the NPC care performance results. Between 2008 and 2012, SJMC’s composite score, a measure of overall performance for NPC care, has been consistently between 96% and 100%. A 96 percent composite score means that SJMC provided an evidence-based NPC cancer treatment 96 times for every 100 opportunities to do so.

Performance for the 5 individual process performance measures (excluding survival outcome) varies from as low as 86% to perfect score of 100%. Perfect 100% score was consistently achieved for the measure “Pathology report confirming malignancy”. The “Radiotherapy for Stage I or II within 90 days of diagnosis” has been consistently between 93% and 100%. While the “Radiotherapy and concurrent Cisplatin/Carboplatin for Stage III, IVA and IVB within 90 days of diagnosis” has been consistently between 86% and 100% and “Radiotherapy and neo-adjuvant Cisplatin/Carboplatin for Stage III, IVA and IVB within 180 days of diagnosis” has been consistently between 88% and 100%.

*Nasopharyngeal Carcinoma survival outcome performance*

**Figure 1:**
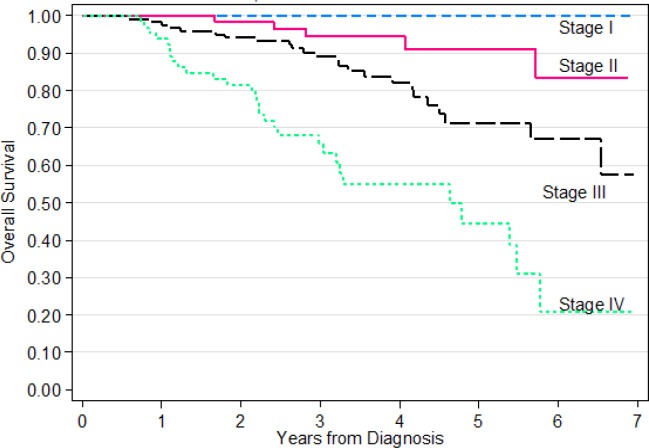
Overall Survival of Patients with Nasopharyngeal Carcinoma Treated at SJMC 2008-2012

**Figure 2:**
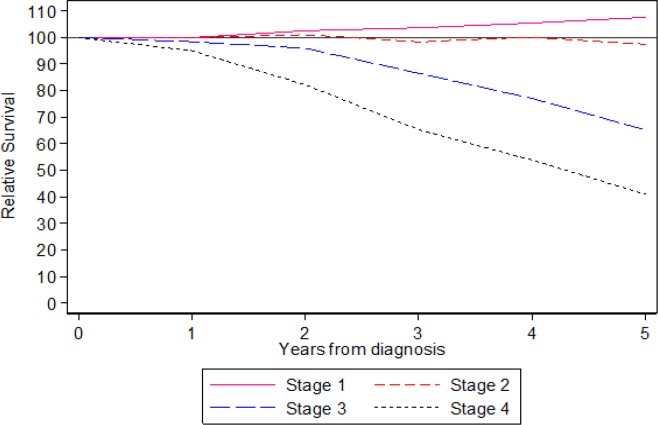
Relative Survival of Patients with Nasopharyngeal Carcinoma Treated at SJMC, 2008-2012

**Figure 3:**
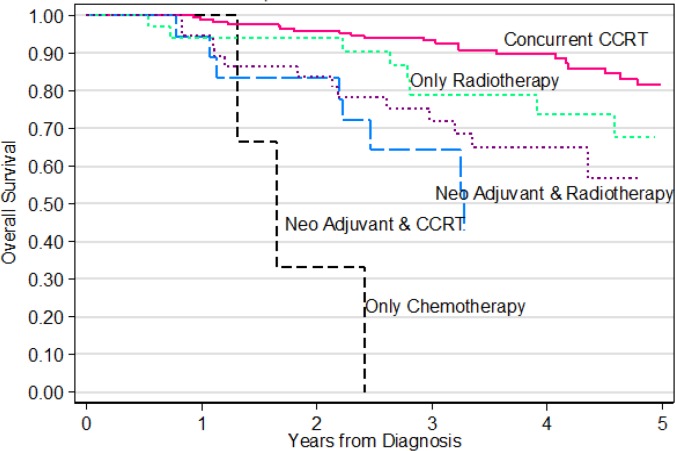
Overall Survival of Patients with Nasopharyngeal Carcinoma Treated at SJMC 2008 -2012 by Treatment Type

Among patients treated at SJMC between 2008 and 2012, the overall survival at 5 years was 100% for patients with Stage I disease, 91% for Stage II disease, 71% for Stage III disease and decreasing to 44% for Stage IV disease. The relative survival at 5 years was 108% for patients with Stage I disease, indicating these patients were practically cured of their cancers. For Stage II disease, the result was 97%. Overall 5 years relative survival was 78.1%. Kaplan-Meier Estimate for Survival Time of NPC Patients are shown in Figures 1, 2 and 3.

As shown in Table 3, univariate analysis of overall survival showed the following factors to be statistically significant: stage (p<0.0001), Treatment Type (p=0.012), Duration from diagnosis to first treatment (p=0.002). However, in the multivariate analysis (Table 3), only Stage remains statistically significant.

## Discussion

Malaysia cancer care performance has been previously reported by Lim et al., (2014). This study noted that performance results are probably acceptable for a middle income country which is consistent with the 95% or higher adherence rates routinely reported by centres in developed countries

*Demographic*

Our centre patients mean age was only 51 years. The study by Ab Hamid et al., (2014) in Kelantan which included 134 patients reported the mean age at diagnosis of NPC patients was 48.12 years old (SD 15.88). Prasad and Rampal, (1992) study in Peninsular Malaysia reported that the mean of 365 newly diagnosed patients was 46.8 years old (SD 12.2). Mak et al., (2015) paper showed that their cohort mean age was 52 years. 

93% of our patients was Chinese. This is likely due to the fact that hospital is situated in an urban area that has more ethnic Chinese population. Prasad and Rampal, (1992) noted that 86.3% were of Chinese origin while Malays constituted 12.9% and Indians make up 0.8% of the NPC patients. Phua et al., (2011) noted that 81.3% were of Chinese origin while Malays constituted 13.6% and Indians make up 1.1% of the NPC patients. Overall, our centre patient demographics reflected NPC disease distribution which is consistent with earlier reports.

As comparison Mak et al., (2015) based on Singapore cohort, reported the distribution of ethnicity was: 476 Chinese (85.3%), 57 Malays (10.2%), and 25 of other ethnic groups (4.5%). Patients of other ethnicities include Indian (6, 1.1%), Caucasian (2, 0.4%), Kenyan (1, 0.2%) and others of mixed ethnicities (16, 2.9%). 

*Stage*

We saw 67% of patients in our cohort presenting at Stage III/IV while only 31% were diagnosed with Early NPC Cancer (Stage I or II). In contrast Phua et al., (2011) centre saw a total of 79.3% of patients presented with stage III or IV disease. In another publication Phua et al., (2013) reported 75.6% with stage 3-4 disease. Ab Hamid et al., (2014) cohort from East Malaysia state presented with 3.0%, 9.8%, 39.1% and 40.6% patients diagnosed at stage I, II, III and IV respectively. In this study 79.7% patients were diagnosed at advanced stage III and IV. According to Colaco et al., (2012) study which focused on UK-based population, Stage III and IV presentation rates were 34 and 38%, respectively. 

The late stage presentation of the disease is consistent with the nature of NPC which usually grow without demonstrating signs and symptoms because of the location and anatomical structure of the nasopharynx (Licitra et al., 2003). As such, continued public education must be sustained to raise awareness of NPC signs and symptoms to allow diagnosis at early stage. Screening measures to pick disease up early is also eagerly awaited.

*Histological Subtype: HPE*

28% of our patients had non-keratinizing differentiated (WHO type II) while 70% presented with non-keratinizing undifferentiated (WHO type III). It was almost similar to the study by Ab Hamid et al., (2014) and Phua et al., (2013) which about 69.1% and 70.5% of the patient presented with WHO III respectively, 

*Treatment*

Concurrent chemo radiation was widely used for patients at SJMC with stage II to Stage IVB disease with more advanced cases receiving neo-adjuvant combination chemotherapy as well. In addition, advanced Intensity Modulated Radiation Therapy (IMRT) with doses up to 70Gy in 35 fractions was used for majority of patients since 2008 and which was further improved by using Tomotherapy Helical IMRT since 2010. IMRT is now the standard of care for NPC in our institution. This concurs with many studies that have found that IMRT when used to allow dose escalation up to 66 Gy and above to the gross tumour volume have shown improved outcomes. (Kwong et al., 2006; Kam et al., 2004). 

99% of our patients received radiotherapy and 88% received radiotherapy with chemotherapy. 12% patients received radiotherapy alone while 65% received concurrent chemo-radiotherapy whereas the remaining 7% patients had neo-adjuvant chemotherapy followed by concurrent chemo-radiotherapy

In Phua et al., (2014) study, Radical RT was given to 162 patients with 22.7% having RT alone and 69.3% having CCRT. Hamid (2014) paper recorded that majority of the patients, 86.5% received radiotherapy followed by 63.9% patients treated with chemotherapy. 

**Table 1:** Patients’ and Tumour Characteristics at Diagnosis, All Patients Diagnosed in Year 2008 to 2012

Patient characteristics	N (%)
Age distribution	
Age<40	39 (15)
Age 40-49	82 (31)
Age 50-59	84 (32)
Age≥60	61 (23)
Sex	
Male	183 (69)
Female	83 (31)
Race	
Malay	11 (4)
Chinese	248 (93)
Indian	0 (0)
Others	7 (3)
Healthcare funding	
Public	0 (0)
Social Security Organization	7 (3)
Non-governmental organization /Charity	0 (0)
Self-funded	119 (45)
Private Insurance	116 (44)
Employers	20 (8)
Others	4 (1)
Location	
Klang Valley & Selangor	148 (56)
Outside Selangor	118 (44)
Origin	
Local	50 (19)
Referral	216 (81)
Stage at diagnosis	
Stage I	22 (8)
Stage II	60 (23)
Stage III	118 (44)
Stage IVA	22 (8)
Stage IVB	33 (12)
Stage IVC	11 (4)
Histology	
WHO I (Keratinizing SCC)	5 (2)
WHO II (Non-keratinizing Differentiated)	74 (28)
WHO III (Non-keratinizing Undifferentiated)	185 (70)
No data	2 (1)

**Table 2:** Summary of Process Performance Measures for Nasopharyngeal Carcinoma Care, SJMC Year 2008 to 2012 of the 266 Cases in This Study

Performance measures	# Eligible*	% Adhered†	Percentage (%)
1. Pathology report confirming malignancy	266	266	100
2. Radiotherapy for Stage I within 90 days of diagnosis	20	19	95
3. Radiotherapy for Stage I or II within 90 days of diagnosis	75	73	97
4. Radiotherapy and concurrent Cisplatin /Carboplatin only for Stage III, IVA and IVB within 90 days of diagnosis	108	105	97
5. All Radiotherapy and Cisplatin/Carboplatin for Stage III, IVA and IVB within 120 days of diagnosis including Neo-adjuvant Chemotherapy ¶	122	118	97
Composite score for above measures			99

*, Number of patients who are eligible for inclusion for this performance analysis; †, Number (%) of eligible patients whose care adhered with the performance measure; ¶, The duration of Radiotherapy was accounted for the Neo adjuvant Chemotherapy.

**Table 3:** Univariate and Multivariate Analyses of Factors Associated with Overall Survival for Patients with Non-Keratinizing Nasopharyngeal Carcinoma Only who Had Received Radiotherapy at SJMC

		Uni-variate		Multi-variate	
Characteristics	Number of patients	Hazard ratio	P-value	Hazard ratio	P-value
Age Group					
<50 years^ref^	117	1		1	
≥50 years	139	1.128	0.661	1.09	0.772
Gender					
Male^ref^	176	1		1	
Female	80	0.863	0.621	1.056	0.859
Ethnicity					
Non-Chinese ^ref^	17	1		1	
Chinese	239	1.1	0.873	0.755	0.647
Stage					
I & II ^ref^	80	1		1	
III	114	3.669	0.009	4.115	0.006
IV	62	12.383	<0.0001	9.405	<0.0001
Histology					
WHO II ^ref^	72	1		1	
WHO III	184	1.233	0.501	1.078	0.823
Treatment Type					
Radiotherapy only ^ref^	32	1		1	
Neo-adjuvant with CCRT*	17	3.587	0.012	1.149	0.814
Neo-adjuvant with RT**	37	2.229	0.086	0.884	0.815
Concurrent CCRT only	170	0.612	0.254	0.492	0.13
Duration of RT**					
≤7 weeks ^ref^ (standard treatment time)	176	1		1	
>7 weeks (prolonged/ interrupted treatment)	80	0.838	0.562	0.786	0.458
Duration from Diagnosis to RT**					
≤ 90 days ^ref^	230	1		1	
> 90 days	26	0.792	0.002	1.175	0.716
Duration from Diagnosis to first treatment					
≤ 30 days ^ref^	191	1		1	
> 30 days	65	1.4	0.251	1.201	0.593

*, Concurrent chemoradiotherapy; **, Radiotherapy

*Survival outcome*

Patients treated at SJMC between 2008 and 2012 had a relative survival at 5-year of 108% for Stage I NPC. This means all such patients were effectively cured by the treatment they had received at SJMC. Even for Stage IV disease, SJMC’s relative survival result was a respectable 44%. SJMC’s relative survival for all stages was 78.1%. The recent Malaysian Cancer Survival Study Report 2018 published by National Cancer Institute, Ministry of Health Malaysia (22), reported 5-year Relative Survival of 46.0%.

Phua et al., (2011, 2013) previously reported the survival rate of a cohort in a public hospital and academic medical centre. Ab Hamid et al., (2014) described the survival outcomes of an east coast public academic medical centre. Centres in Singapore (Mak et al., 2015), China (Sun et al., 2014) Taiwan (Huang et al., 2015) and Hong Kong has reported various survival rates as well. 

Phua et al., (2013) found that the survival according to stage was 81.8% for stage I, 77.9% for stage II, 47.4% for stage III and 25.9% for stage IV. Later in 2014 , Ab Hamid et al., (2014) from Hospital University Sains Malaysia reported their five-year survivals according to stage I, II, III, IV were 50.0%, 66.0 %, 41.8% and 28.0% respectively. Huang et al., (2015) reported their 1-, 2-, 5- and 8-year overall survival (OS) rates as 89.6%, 80.4%, 65.2% and 56.5%, respectively.

SJMC’s result for overall survival at 5 years for all stages was 73%, better than the 65% survival reported by GLOBOCAN for Malaysia in 2012 (IARC, 2013). SJMC’s stage specific survival at 5 years (100%, 91%, 71% and 44% for Stage I, II, III and IV respectively) were similarly much better than those reported by individual public and university centres (mean 73%, 73%, 49% and 24% for Stage I, II, III and IV respectively) in Malaysia (Phua et al., 2011; Phua et al., 2014; Ab Hamid et al., 2014). 

*These differences could be attributed to several reasons:*

a. Earlier presentation of the disease as 75.6% of Phua (2014) cohort presented with Stage III/IV while 67% of SJMC cohort presented in these stages. This could explain differences in overall survival but NOT stage specific survival 

b. Access to IMRT. 92% of SJMC patients received IMRT while Phua(2013) reported all patients were treated with 3DCRT with no patients receiving IMRT. 

c. Diagnostic and Treatment delay. In SJMC, the median duration from presentation to diagnosis was 7 days, and median duration to initiation of treatment after diagnosis 21 days. Median duration to initiate any treatment for Stage III/IV was only 21 days while for Stage I/II was 23 days. Median Duration from diagnosis to starting Radiotherapy for advanced disease was 17 days with a mean of 24 days

d. Other patients’ level confounders such as age, socio-economic status

In comparison, a Singapore study reported 5-year overall survival (OS) rates of their cohort at 69.9%. The overall survival results at 5 years for Singapore, Hong Kong and Taiwan as reported by GLOBOCAN in 2012 were 56%, 62% and 47% respectively (IARC, 2013).

Our study indicated that Stage at Diagnosis was a significant prognostic factor for Overall Survival. This is similar to earlier studies (El-Sherbieny et al.,2011; Mak HW et al., 2015)

In conclusion SJMC is among the first hospitals in Malaysia to embark on routine measurement of the performance of its cancer care services. SJMC’s NPC care process performance results between 2008 and 2012 have been consistently between 96% and 100%, close to or better than the benchmark of 95%. Patients treated at SJMC between 2008 and 2012 had a relative survival at 5-year of 108% for Stage I NPC. This means all such patients were cured by the treatment they had received at SJMC. Even for Stage IV disease, SJMC’s relative survival result was a respectable 44%

Continued public education must be sustained to raise awareness of NPC signs and symptoms to allow diagnosis at early stage. 

## Funding Statement

This study was funded by Subang Jaya Medical Centre

## Statement conflict of interest

There is no conflict of interest
